# Predicting risk factors for rebleeding, infections, mortality following peptic ulcer bleeding in patients with cirrhosis and the impact of antibiotics prophylaxis at different clinical stages of the disease

**DOI:** 10.1186/s12876-015-0289-z

**Published:** 2015-05-20

**Authors:** Ming-Te Kuo, Shih-Cheng Yang, Lung-Sheng Lu, Chien-Ning Hsu, Yuan-Hung Kuo, Chung-Huang Kuo, Chih-Ming Liang, Chung-Mou Kuo, Cheng-Kun Wu, Wei-Chen Tai, Seng-Kee Chuah

**Affiliations:** Division of Hepatogastroenterology, Department of Internal Medicine, Kaohsiung Chang Gung Memorial Hospital, Chang Gung University College of Medicine, Kaohsiung, Taiwan; Department of Pharmacy, Kaohsiung Chang Gung Memorial Hospital and Chang Gung University; College of Medicine, Kaohsiung, Taiwan; Division of Hepatogastroenterology, Kaohsiung Chang Gang Memorial Hospital, 123 Ta-Pei Road, Niaosung Hsiang, Kaohsiung City, 833 Taiwan

**Keywords:** Liver cirrhosis, Peptic ulcer bleeding, Antibiotic prophylaxis, Bacterial infections, Rebleeding, In-hospital mortality

## Abstract

**Background:**

Infections in cirrhotic patients with upper gastrointestinal bleeding are a common event causing severe complication and mortality. This study aimed to identify risk factors that may predict rebleeding, bacterial infections, and the impact of antibiotic prophylaxis on mortality at different stages of cirrhosis following acute peptic ulcer bleeding (PUB).

**Methods:**

A hospital-based retrospective cohort study was conducted on 235 cirrhotic patients with acute peptic ulcer hemorrhage who underwent therapeutic endoscopic procedures between January 2008 and January 2014 (*n* = 235); of these, 88 patients received prophylactic intravenous ceftriaxone (antibiotic group) and 147 patients did not (nil-antibiotic group). The recorded outcomes were length of hospital stay, bacterial infection, rebleeding, and in-hospital mortality.

**Results:**

Forty-eight (20.4 %) patients experienced ulcer rebleeding and 46 (19.6 %) developed bacterial infections. More patients suffered from infection and recurrent bleeding in the nil-antibiotic group than the antibiotic group (25.2 % vs. 10.2 %, *p* = 0.005 and 30.6 % vs. 3.4 %; p < 0.001, respectively). The predictive risk factors for rebleeding were the Rockall score (*p* = 0.004), units of blood transfusion (*p* = 0.031), and no antibiotic prophylaxis (p <0.001); for bacterial infections, they were the Child-Pugh score (*p* = 0.003), active alcoholism (*p* = 0.035), and no antibiotic prophylaxis (*p* = 0.009). Overall, 40 (17 %) patients died during hospitalization. The Rockall score and rebleeding were predictive factors for in-hospital mortality. In subgroup analysis, survival was significantly reduced in decompensated patients (*p* = 0.034).

**Conclusions:**

This study suggests that antibiotic prophylaxis after endoscopic hemostasis for acute PUB prevented infections and reduced rebleeding events in cirrhotic patients. Antibiotic prophylaxis improved survival among decompensated cohort following PUB. The Rockall score and rebleeding were predictive risk factors for in-hospital mortality.

## Background

Upper gastrointestinal bleeding in cirrhotic patients is associated with an increased rate of failure to control bleeding and can result in mortality when bacterial infections occur [1, 2]. This is evidenced by reports demonstrating that oral administration of non-absorbable antibiotics markedly reduces the incidence of bacterial infections in cirrhotic patients with gastrointestinal hemorrhage [3]. Patients with cirrhosis are at risk for both variceal and nonvariceal causes of upper gastrointestinal bleeding; however, only variceal bleeding has been widely studied. Nevertheless, there remains one third of nonvariceal upper gastrointestinal bleeding in cirrhotic patients due to peptic ulcers (PU) as reported in the literature [4]. In addition, bleeding PU have been associated with substantial morbidity and mortality in cirrhotic patients [5]. A multicenter prospective study from Italy reported that 10 % of patients with cirrhosis rebleed and 15 % of them died within 6 weeks after acute nonvariceal upper gastrointestinal bleeding [5].

Bacterial infection is more prone to initiate a systemic inflammatory response syndrome and increases the probability of death in patients with decompensated cirrhosis [6]. Generally, cirrhotic patients with PUB become infected and rebleed more frequently compared to non-cirrhotic patients [4]. To our knowledge, prognosis after treating acute PUB has not been well studied in cirrhotic patients. Most studies on antibiotic prophylaxis in cirrhotic patients have focused on those with variceal hemorrhages [7–9]. Therefore, we conducted this study to identify the risk factors that predict re-bleeding, bacterial infections, mortality and the impact of antibiotic prophylaxis on death at different stages of cirrhosis following acute PUB.

## Methods

### Ethics statements

This retrospective chart review study was approved by both the Institutional Review Board and Ethics Committee of Chang Gung Memorial Hospital, Taiwan (IRB103-5018B). The Ethics Committee waived the requirement for informed consent, and each patient’s medical records was anonymized and de-identified prior to access. All patients provided their written inform consent before endoscopic interventions.

### Patient population

This was a hospital-based retrospective cohort study including 426 cirrhotic eligible patients with PUB who underwent endoscopic interventions over a 6 year period between January 2008 and January 2014. The following patients were excluded from the study: (1) presence of any of the following signs of infection; fever >38 °C, white blood cell count >10 000/mm^3^, immature neutrophils >500/mm^3^, a polymorphonuclear cell count in ascitic fluid >250/mm^3^, ≥15 leukocytes/field in the fresh urine sediment, or data compatible with pneumonia on the chest X-ray (*n* = 105), (2) allergy to cephalosporins (*n* = 2), (3) patients who received oral/parenteral antibiotics in the week prior to the procedure (*n* = 18), (4) a source of upper gastrointestinal bleeding other than PUB (*n* = 37), (5) patients who died within the first day after admission (*n* = 3), (6) patients who did not complete the in-hospital follow-up period (*n* = 26). A total of 235 patients were recruited into current study (male/female: 181/54; mean age: 62.2 ± 13.6 y). Those patients who received prophylactic intravenous infusion of ceftriaxone at 1 g per 12 h were classified as the antibiotic group (*n* = 88) in contrast to the control group who did not receive antibiotics (*n* = 147). The antibiotic group has been routine practice to prescribe prophylactic antibiotics to cirrhotic patients with gastrointestinal bleeding in our hospital since the year 2010 according to guidelines. On the other hand, those patients belonged to control group attended the hospital between January 2008 and December 2009. The antibiotic prophylaxis was given immediately after patients receiving endoscopic treatment and two sets of blood culture were obtained before administering the antibiotics even these patients did not encounter infections according to our hospital policy.

PUB was defined by the existence of signs of hematemesis, coffee ground vomitus, hematochezia, or melena and proven high-risk ulcers by endoscopy that were defined according to the Forrest classification [10]. High risk bleeding stigmata were referred for endoscopic view of active bleeding, visible vessels, or adherent clots. Patients’ performance status was stratified according to the Rockall classification [11]. Blood transfusions were given to maintain hematocrit levels between 25 % and 30 %. Patients with PUB were treated with intravenous high dose pantoprazole (80 mg intravenous bolus followed by 200 mg continuous infusion for three days).

### Data collection and analysis

The first authors abstracted following information from patient records (including written charts and electronic data sources). All patients had a complete medical assessment at initial hospital admission, including documentation of clinical, biochemical, and endoscopic factors that might contribute to rebleeding and mortality. The registered clinical variables were demographic data, clinical manifestations of bleeding, and the use of tobacco, alcohol, aspirin, clopidogrel, and non-steroidal anti-inflammatory drugs (NSAID); co-morbidities such as diabetes mellitus, cardiovascular disease, stroke, end-stage renal disease, and chronic pulmonary disease were analyzed. Other clinical characteristics such as age, sex, and hemodynamic instability on admission and laboratory data such as white blood cell count, hemoglobin, platelet count, prothrombin time, serum creatinine, serum albumin, and total bilirubin were analyzed. The recorded endpoints were occurrence of infection, rebleeding, length of hospital stay, and in-hospital death.

### Definitions

Time to endoscopy was defined as the time interval from admission via the emergency department to initial endoscopy, expressed in hours. The diagnosis of liver cirrhosis was based on clinical, laboratory, abdominal ultrasonographic, or histological findings [12]. Decompensated liver cirrhosis was used to describe patients complicated by ascites, jaundice (bilirubin level >3.0 mg/dl), and a history of identified varices or hepatic encephalopathy. Diagnosis of hepatitis C and B virus-related liver disease was determined with specific viral markers (HBsAg or anti-HCV). Alcohol-related liver disease was defined as daily alcohol consumption >80 g in men and >40 g in women for at least 10 years with negative viral, metabolic, and autoimmune markers [13]. Active alcoholism is defined as a continuing daily alcohol intake over 20 g in patients with alcoholic cirrhosis [13].

Rebleeding was defined as a new onset of hematemesis, melena, or both associated with tachycardia or hypovolemic shock or a decrease in serum hemoglobin level of >2 g/dl after successful endoscopic and pharmacological treatment and hemodynamic stability of at least a 24 h period of stable vital signs [14].

A diagnosis of bacteremia was made when the presence of viable bacteria in the blood and the clinical picture was consistent with this diagnosis. The diagnosis of spontaneous bacterial peritonitis was made when a positive culture of ascitic fluid was obtained with an ascitic fluid neutrophil count ≥250 neutrophils/μl [15]. The diagnosis of pneumonia was made by clinical, radiological, and bacteriological data. The diagnosis of urinary tract infection was made when a positive culture of urine (10^5^ ≥ colonies/ml) was obtained with a urine neutrophil count >10 neutrophils/μl and associated clinical pictures.

### Statistical analysis

All results were expressed as means ± standard deviations for continuous variables and as relative frequencies or percentages for categorical variables. Distributions of continuous variables were analyzed by the X^2^ test, the Fisher’s exact test, or the independent sample *t*-test, depending upon the type of data analyzed for the two groups where appropriate. The Kaplan–Meier analysis with the log-rank test was used to compare differences of death rates between the two groups. Variables were analyzed using the multivariate Cox proportional hazard model to determine independent predictive factors of infection, rebleeding, and mortality. All the variables in univariate analyses were analyzed in multivariate analyses. The results were expressed as a hazard ratio (HR) with 95 % confidence intervals. All statistical analyses were performed using the SPSS v18.0 (Chicago, Illinois, USA). A *p*-value of <0.05 was considered statistically significant.

## Results

All the patients’ baseline characteristics are presented in Table 1. All patients underwent emergency endoscopy within 24 h of admission. The mean time from presentation of bleeding to endoscopy was 7.6 ± 7 h. One hundred and forty-six patients (62.1 %) suffered from bleeding gastric ulcers (GU), 73 (31.1 %) from duodenal ulcers (DU), and 16 (6.8 %) from both GU and DU. These ulcers had high risk stigmata of hemorrhage (Forrest Ia or Ib, 50.2 % and Forrest II a or IIb, 38.7 %). Among them, 57.4 % were found to have varices without stigmata of recent hemorrhage [esophageal varices (EV): 51.4 %; gastric varices (GV): 0.9 %; combined EV/GV: 5.1 %]. The mean value of the Rockall scoring system was 4.7 ± 1.6 on admission.

**Table 1:** Clinical characteristics, endoscopic findings, and clinical outcome of cirrhotic patients with peptic ulcer bleeding (*n* = 235)

Characteristics	Antibiotic group (*n* = 88)	Control group (*n* = 147)	*p*-value
Age (y)	61.8 ± 15.2	62.5 ± 12.5	0.699
Male, n (%)	71 (80.7)	110 (74.8)	0.302
Etiology of liver cirrhosis			
Alcoholic, n (%)	24 (27.3)	37 (25.2)	0.722
Viral hepatitis, n (%)	62 (70.4)	100 (68.0)	0.697
Cryptogenic, n (%)	2 (2.3)	10 (6.8)	0.219
Child-Pugh group			
A, n (%)	40 (45.5)	68 (46.3)	0.905
B, n (%)	31 (35.2)	44 (29.9)	0.399
C, n (%)	17 (19.3)	35 (23.8)	0.422
Decompensated cirrhosis, n (%)	44 (50.0)	61 (41.5)	0.204
Rockall score	4.7 ± 1.5	4.7 ± 1.7	0.885
MELD score	13.7 ± 6.1	14.1 ± 6.0	0.631
Child-Pugh score	6.9 ± 1.5	7.1 ± 1.7	0.467
Gastroesophageal varices, n (%)	51 (58.0)	84 (57.1)	0.903
Ulcer location			
Gastric ulcer, n (%)	55 (62.5)	91 (61.9)	0.927
Duodenal ulcer, n (%)	27 (30.7)	46 (31.3)	0.922
Both, n (%)	6 (6.8)	10 (6.8)	1
Use of NSAID or aspirin/clopidogrel, n (%)	17 (19.3)	20 (13.6)	0.245
Smoking, n (%)	33 (37.5)	63 (42.9)	0.419
Active alcoholism, n (%)	31 (35.2)	55 (37.4)	0.736
Other comorbidities			
Diabetes mellitus, n (%)	31 (35.2)	40 (27.2)	0.251
Hypertension, n (%)	39 (44.3)	54 (36.7)	0.311
Cardiovascular disease, n (%)	8 (9.1)	7 (4.8)	0.299
Stroke, n (%)	3 (3.4)	10 (6.8)	0.381
ESRD, n (%)	3 (3.4)	13 (8.8)	0.471
COPD, n (%)	8 (9.1)	7 (4.8)	0.269
Laboratory on admission			
WBC (10^9^/l)	6094.3 ± 2045.3	5876.2 ± 1970.4	0.419
Hb (g/dl)	8.7 ± 2.5	8.9 ± 1.9	0.437
PLT (10^9^/l)	124.5 ± 60.5	121.5 ± 71.3	0.731
Prothrombin time (s)	13.6 ± 4.8	12.4 ± 2.7	0.031
Albumin (g/dl)	3.0 ± 0.7	2.8 ± 0.7	0.152
Creatinine (mg/dl)	1.6 ± 2.0	2.0 ± 2.5	0.126
Total bilirubin (mg/dl)	3.0 ± 3.8	3.1 ± 3.8	0.961
Clinical characteristics			
Hypovolemic shock on admission, n (%)	8 (9.1)	13 (8.8)	0.949
Blood units transfused (unit)	4.8 ± 5.6	5.2 ± 7.6	0.637
Stigmata of recent hemorrhage at ulcer			
Forrest Ia or Ib ulcer, n (%)	44 (50)	74 (50.3)	0.960
Forrest IIa or IIb ulcer, n (%)	37 (42)	54 (36.7)	0.419
Forrest IIc ulcer, n (%)	7 (8)	19 (13)	0.240
Time (h), bleeding to endoscopic treatment	6.9 ± 6.7	7.7 ± 6.6	0.382
Treatment			
Epinephrine injection, n (%)	27 (30.7)	57 (38.8)	0.261
APC, n (%)	13 (14.8)	37 (25.2)	0.070
Hemoclipping, n (%)	14 (15.9)	6 (4.1)	0.003
Combined therapy, n (%)	34 (38.6)	47 (32.0)	0.323
Outcomes			
Hospital stay (d)	15.7 ± 13.1	14.1 ± 10.9	0.282
Rebleeding, n (%)	3 (3.4)	45 (30.6)	<0.001
Infections, n (%)	9 (10.2)	37 (25.2)	0.005
In-hospital mortality, n (%)	12 (13.6)	28 (19.0)	0.112
Failure to control bleeding, n (%)	1 (1.1)	7 (4.8)	
Sepsis, n (%)	3 (3.4)	7 (4.8)	
Multiple organ failure, n (%)	8 (9.1)	14 (9.4)	

Abbreviations: *APC*, argon plasma coagulation; *COPD*, chronic obstructive pulmonary disease; *ESRD*, end-stage renal disease; *EV*, esophageal varices; *GOV*, Gastroesophageal varices; *IGV*, Isolated gastric varices; *MELD*, Model for End-Stage Liver Disease; *NSAID*, nonsteroidal anti-inflammatory drug; *WBC*, white blood cells; *Hb*, hemoglobin; *PLT*, platelet count; *PT*, prothrombin time

The details of endoscopic interventions are also summarized in Table 1. Endoscopic intervention was performed on all patients with either monotherapy (65.5 %) or combination therapy (34.5 %). All patients received intravenous high dose proton pump inhibitors for 3 days after initial endoscopic hemostasis. The mean length of hospital stay was 14.9 ± 11.6 d. Patients in the antibiotic group had a similar hospital stay when compared with control patients (*p* = 0.282).

Overall, 48 (20.4 %) patients encountered rebleeding events. Rebleeding rate in the antibiotic group was significantly lower than the control group (3.4 % vs 30.6 %; p <0.001; Table 1). On multivariate analysis, the risk factors for rebleeding were higher rockall score [HR = 1.069; 95 % confidence interval (CI) = 1.022–1.120; *p* = 0.004] and more units of blood transfusion (HR = 1.019; 95 % CI = 1.002–1.037; *p* = 0.031). Antibiotic prophylaxis has a protective role for rebleeding in these patients (HR = 0.082; 95 % CI = 0.025–0.267; p <0.001) (Table 2). It is interesting that time to endoscopy, high risk bleeding stigmata, and endoscopic therapeutic methods were not associated with rebleeding rate (Table 2).

**Table 2:** Univariate and multivariate analysis of potential risk factors for rebleeding in patients with peptic ulcer bleeding after endoscopic treatment

Variable	Univariate analysis		Multivariate analysis	
	Hazard ratio (95 % CI)	*p*-value	Hazard ratio (95 % CI)	*p*-value
Age	0.991 (0.971–1.012)	0.383		
Male gender	2.882 (1.141–7.283)	0.025	2.639 (0.968–7.198)	0.058
Etiology of liver cirrhosis				
Alcoholic	1.001 (0.521–1.924)	0.998		
Viral hepatitis	1.086 (0.790–1.494)	0.610		
Cryptogenic	0.715 (0.369–1.384)	0.319		
Decompensated cirrhosis	1.559 (0.881–2.758)	0.127		
Rockall score	1.071 (1.031–1.111)	<0.001	1.069 (1.022–1.120)	0.004
MELD score	1.141 (0.964–1.351)	0.126		
Child-Pugh score	1.321 (1.126–1.548)	0.001	1.128 (0.923–1.378)	0.238
Other comorbidities				
Diabetes mellitus	1.244 (0.689–2.249)	0.469		
Hypertension	0.688 (0.378–1.255)	0.233		
CVD	0.948 (0.230–3.912)	0.941		
Stroke	1.360 (0.422–4.382)	0.607		
ESRD	1.246 (0.448–3.470)	0.673		
COPD	0.495 (0.145–1.683)	0.260		
Use of NSAID or aspirin	1.479 (0.586–3.734)	0.408		
Smoking	1.362 (0.771–2.405)	0.287		
Active alcoholism	1.585 (0.898–2.799)	0.112		
WBC (10^9^/l)	1	0.886		
Hb (g/dl)	0.990 (0.869–1.128)	0.880		
PLT (10^9^/l)	0.997 (0.992–1.002)	0.260		
PT (s)	1.286 (0.749–2.207)	0.362		
Creatinine (mg/dl)	1.063 (0.966–1.171)	0.213		
Albumin (g/l)	0.635 (0.402–1.005)	0.053		
Total bilirubin	1.068 (1.012–1.128)	0.017	1.042 (0.967–1.122)	0.277
Blood units transfused	1.032 (1.015–1.050)	<0.001	1.019 (1.002–1.037)	0.031
Hypovolemic shock on admission	1.143 (0.453–2.887)	0.777		
High risk bleeding stigmata at ulcer base	1.103 (0.407–2.989)	0.848		
Time (h), bleeding to endoscopic treatment	0.989 (0.959–1.039)	0.940		
Combined treatment of endoscopic hemostasis	0.941 (0.521–1.700)	0.840		
Bacterial infection	1.974 (1.081–3.607)	0.027	1.012 (0.527–1.941)	0.972
Antibiotic prophylaxis	0.090 (0.028–0.291)	<0.001	0.082 (0.025–0.267)	<0.001

Abbreviations: *CI*, confidence interval; *COPD*, chronic obstructive pulmonary disease; *CVD*, Cardiovascular disease; *ESRD*, end-stage renal disease; *MELD*, Model for End-Stage Liver Disease; *NSAID*, nonsteroidal anti-inflammatory drug; *WBC*, white blood cells; *Hb*, hemoglobin; *PLT*, platelet count; *PT*, prothrombin time

Bacterial infections were documented in 46 patients (19.6 %); more patients were infected in the control group (10.2 % vs. 25.2 %; *p* = 0.005). Infections were confirmed for 9 patients in the antibiotics group (bacteremia in 4, spontaneous bacterial peritonitis in 2, pneumonia in 1 and urinary tract infection in 2). On the other hand, 37 bacterial infections were proven in the control group (bacteremia in 17, pneumonia in 4, spontaneous bacterial peritonitis in 10, and urinary tract infections in 6). The causative organisms of bacteremia were gram-negative bacilli in 15 patients (*Klebsiella pneumoniae* in 7; *Escherichia coli* in 6; *Pseudomonas aeruginosa* in 2) and gram-positive cocci in 6 patients (*Streptococcus pneumoniae* in 4; *Staphylococcus aureus* in 1; *Enterococcus faecalis* in 1). The risk factors for bacterial infection were higher Child-Pugh score (HR = 1.251; 95 % CI = 1.080–1.449; *p* = 0.003) and active alcoholism (HR = 1.882; 95 % CI = 1.045–3.388; *p* = 0.035). Antibiotic prophylaxis played a significant role to prevent infections (HR = 0.377; 95 % CI = 0.180–0.786; *p* = 0.009) (Table 3).

**Table 3:** Univariate and multivariate analysis of potential risk factors for infection in patients with peptic ulcer bleeding after endoscopic treatment

Variable	Univariate analysis		Multivariate analysis	
	Hazard ratio (95 % CI)	*p*-value	Hazard ratio (95 % CI)	*p*-value
Age	0.989 (0.968–1.010)	0.286		
Male gender	1.171 (0.580–2.363)	0.660		
Etiology of liver cirrhosis				
Alcoholic	1.869 (1.015–3.441)	0.045	1.842 (0.899–3.774)	0.095
Viral hepatitis	0.769 (0.572–1.035)	0.083		
Cryptogenic	0.933 (0.582–1.498)	0.775		
Decompensated cirrhosis	2.339 (1.273–4.298)	0.006	1.688 (0.707–4.043)	0.239
Rockall score	1.275 (1.048–1.552)	0.015	1.141 (0.944–1.381)	0.173
MELD score	1.061 (1.022–1.100)	0.002	1.048 (0.988–1.111)	0.121
Child-Pugh score	1.289 (1.113–1.493)	0.001	1.251 (1.080–1.449)	0.003
Other comorbidities				
Diabetes mellitus	1.272 (0.693–2.338)	0.437		
Hypertension	0.971 (0.537–1.757)	0.923		
CVD	0.483 (0.066–3.510)	0.472		
Stroke	0.412 (0.057–2.990)	0.380		
ESRD	1.661 (0.402–6.860)	0.483		
COPD	1.981 (0.837–4.686)	0.120		
Use of NSAID or aspirin	0.127 (0.018–0.925)	0.042	0.182 (0.025–1.336)	0.094
Smoking	1.301 (0.725–2.335)	0.378		
Active alcoholism	1.924 (1.075–3.446)	0.028	1.616 (0.789–3.310)	0.190
WBC (10^9^/l)	1	0.804		
Hb (g/dl)	0.899 (0.775–1.019)	0.090		
PLT (10^9^/l)	0.996 (0.991–1.001)	0.160		
PT (second)	1.290 (0.781–2.129)	0.319		
Creatinine (mg/dl)	0.958 (0.830–1.106)	0.562		
Albumin (g/l)	0.921 (0.592–1.434)	0.716		
Total bilirubin	1.071 (1.019–1.127)	0.008	1.006 (0.929–1.088)	0.887
Blood units transfused	1.016 (0.993–1.0140	0.181		
Hypovolemic shock on admission	1.328 (0.525–3.362)	0.550		
High risk bleeding stigmata at ulcer base	2.830 (0.686–11.68)	0.150		
Time (h), bleeding to endoscopic treatment	1.026 (0.997–1.055)	0.077		
Combined treatment of endoscopic hemostasis	0693 (0.385–1.248)	0.222		
Recurrent bleeding	1.974 (1.075– 3.623)	0.028	1.601 (0.781–3.282)	0.906
Antibiotic prophylaxis	0.346 (0.167–0.719)	0.004	0.377 (0.180–0.786)	0.009

Abbreviations: *CI*, confidence interval; *COPD*, chronic obstructive pulmonary disease; *CVD*, Cardiovascular disease; *ESRD*, end-stage renal disease; *MELD*, Model for End-Stage Liver Disease; *NSAID*, nonsteroidal anti-inflammatory drug; *WBC*, white blood cells; *Hb*, hemoglobin; *PLT*, platelet count; *PT*, prothrombin time

In-hospital death occurred in 40 patients (17 %). Causes of death were hypovolemic shock in 8 patients (1 in the antibiotic group and 7 in control group), sepsis in 10 patients (3 and 7 patients, respectively) and multiple organ failure in 22 patients (8 and 14 patients, respectively). There was no significant difference in mortality during hospitalization between patients treated with intravenous ceftriaxone (*n* = 12, 13.6 %) and those in the control group (*n* = 28, 19 %). The observed survival was virtually identical for both groups (*p* = 0.112; Table 1). The results of the univariate and multivariate analyses for independent risks of death after acute PUB are summarized in Table 4. The results of univariate analysis showed that decompensated cirrhosis, total bilirubin level, Rockall score, MELD score, Child-Pugh score, bacterial infection, and recurrent bleeding were associated with an increased risk of death. In multivariate analysis, the in-hospital mortality was remarkably dependent on Rockall score (HR = 1.884; 95 % CI = 1.477–2.404; p <0.001) and recurrent bleeding (HR = 2.796; 95 % CI = 1.473–5.306; *p* = 0.002).

**Table 4:** Univariate and multivariate analysis of potential risk factors for death in patients with peptic ulcer bleeding after endoscopic treatment

Variable	Univariate analysis		Multivariate analysis	
	Hazard ratio (95 % CI)	*p*-value	Hazard ratio (95 % CI)	*p*-value
Age	0.981 (0.958–1.004)	0.112		
Male gender	2.046 (0.902–4.639)	0.087		
Etiology of liver cirrhosis				
Alcoholic	1.188 (0.577–2.455)	0.641		
Viral hepatitis	1.023 (0.722–1.488)	0.887		
Cryptogenic	0.713 (0.366 –1.392)	0.322		
Decompensated cirrhosis	2.112 (1.014–4.402)	0.046	1.259 (0.497–3.188)	0.627
Rockall score	1.638 (1.327–2.022)	<0.001	1.884 (1.477–2.404)	<0.001
MELD score	1.049 (1.007–1.093)	0.023	0.945 (0.875–1.020)	0.417
Child-Pugh score	1.321 (1.126–1.548)	0.001	1.252 (0.915–1.712)	0.163
Other comorbidities				
Diabetes mellitus	1.163 (0.825–1.639)	0.388		
Hypertension	0.826 (0.437–1.562)	0.555		
CVD	1.656 (0.396–6.924)	0.490		
Stroke	0.830 (0.194–3.542)	0.801		
ESRD	1.462 (0.445–4.808)	0.532		
COPD	0.495 (0.145–1.683)	0.260		
Use of NSAID or aspirin	1.381 (0.765–2.493)	0.284		
Smoking	1.040 (0.739–1.465)	0.820		
Active alcoholism	1.453 (0.771–2.739)	0.248		
WBC (10^9^/l)	1	0.737		
Hb (g/dl)	0.941 (0.809–1.095)	0.431		
PLT (10^9^/l)	0.997 (0.992–1.002)	0.211		
PT (second)	1.355 (0.823–2.232)	0.232		
Creatinine (mg/dl)	1.003 (0.857–1.174)	0.972		
Albumin (g/l)	0.906 (0.555–1.479)	0.692		
Total bilirubin	1.056 (1.004–1.111)	0.033	0.987 (0.916–1.063)	0.731
Blood units transfused	0.989 (0.961–1.018)	0.468		
Hypovolemic shock on admission	1.184 (0.420–3.341)	0.750		
High risk bleeding stigmata at ulcer base	1.103 (0.407–2.989)	0.848		
Time (h), bleeding to endoscopic treatment	0.972 (0.936–1.010)	0.143		
Combined treatment of endoscopic hemostasis	0.966 (0.485–1.926)	0.922		
Bacterial infection	1.770 (0.945–3.318)	0.075		
Recurrent bleeding	2.412 (1.292–4.501)	0.006	2.796 (1.473–5.306)	0.002
Antibiotic prophylaxis	0.582 (0.293–1.151)	0.121		

Abbreviations: *CI*, confidence interval; *COPD*, chronic obstructive pulmonary disease; *CVD*, Cardiovascular disease; *ESRD*, end-stage renal disease; *MELD*, Model for End-Stage Liver Disease; *NSAID*, nonsteroidal anti-inflammatory drug; *WBC*, white blood cells; *Hb*, hemoglobin; *PLT*, platelet count; *PT*, prothrombin time

### Subgroup analytical result for compensated and decompensated patients

**Fig. 1:**
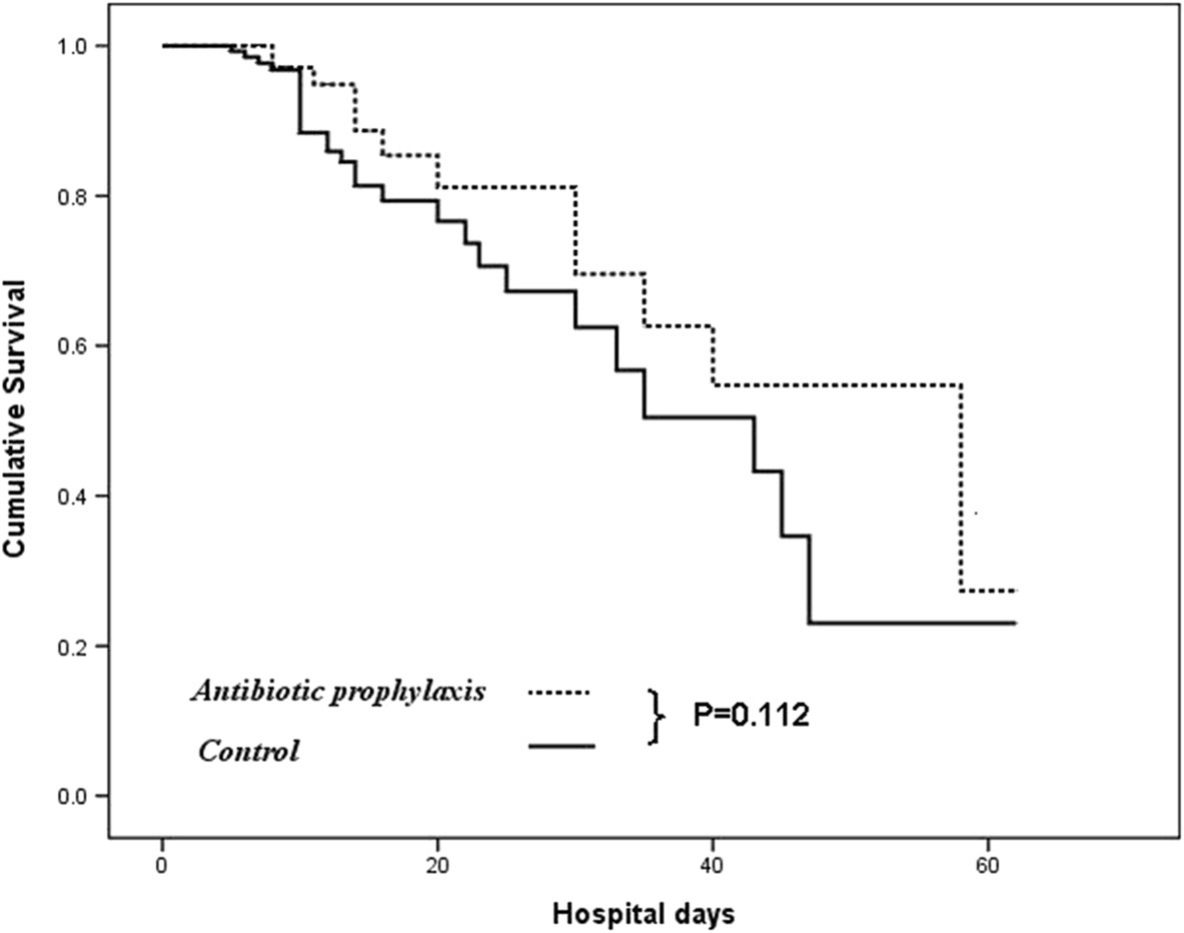
Actuarial probability of remaining survival in all cirrhotic patients after endoscopic interventions for the ceftriaxone group (antibiotic prophylaxis group) and the nil- antibiotic prophylaxis group (control group)

**Fig. 2:**
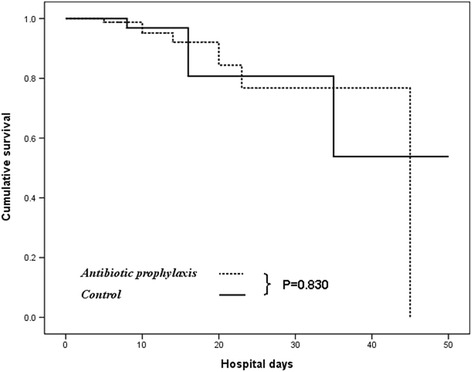
Actuarial probability of remaining survival at different stages of cirrhotic patients (compensated liver cirrhosis). There was a similar probability of survival between compensated patients who were prescribed with intravenous ceftriaxone and those without antibiotic prophylaxis (*p* = 0.830 by log-rank test)

**Fig. 3:**
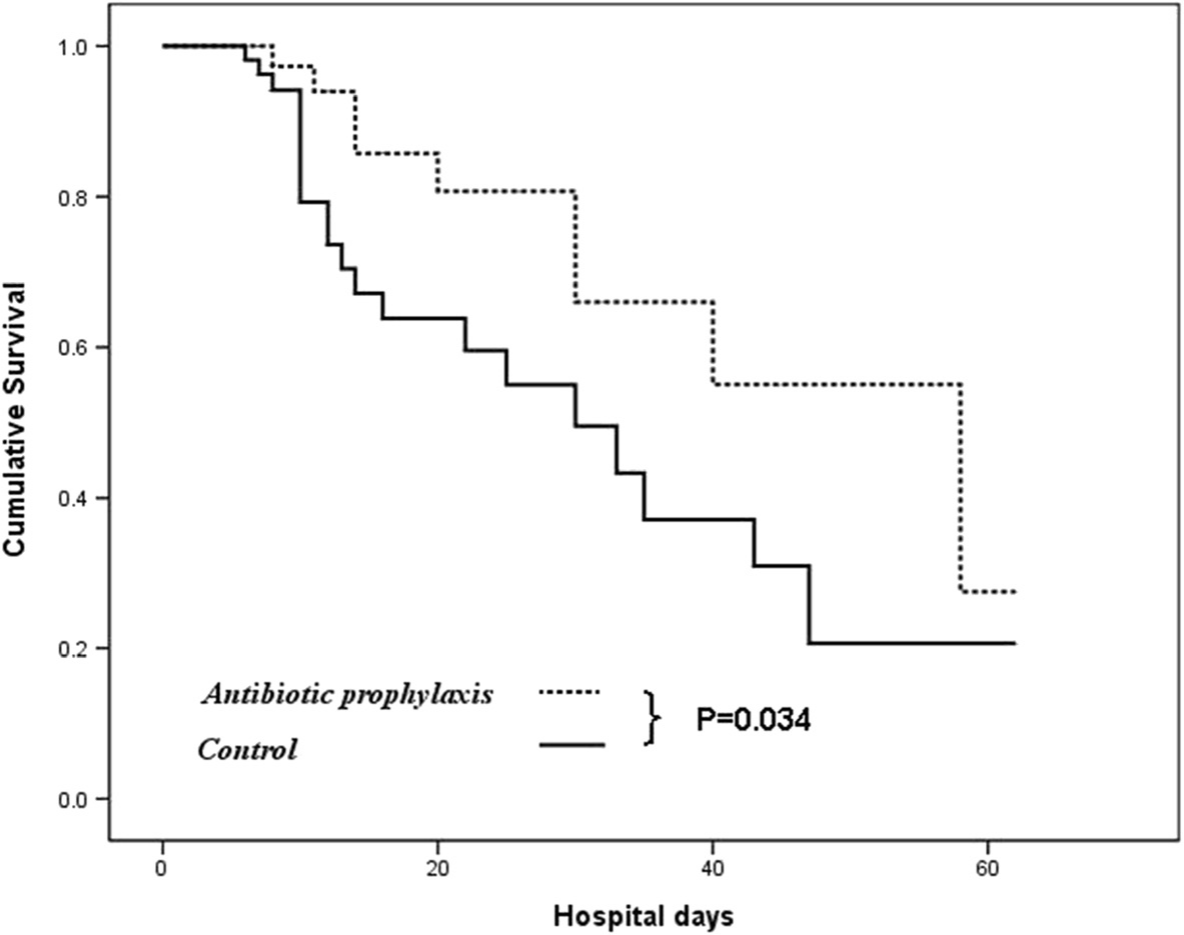
Actuarial probability of remaining survival at different stages of cirrhotic patients (decompensated liver cirrhosis). A significantly higher probability of remaining survival was observed in those who were prescribed with intravenous ceftriaxone than those without antibiotic prophylaxis (*p* = 0.034 by log-rank test)

By using the Kaplan–Meier approach, the administration of prophylactic antibiotics was not associated with significant differences in in-hospital mortality between our cohort (13.6 % vs. 19 %, *p* = 0.112 by log-rank test; Fig. 1). On the other hand, we observed that the in-hospital mortality was 28.6 % in patients with decompensated cirrhosis and 7.7 % in patients with compensated cirrhosis (p <0.001) following acute PUB. Because the lack of a beneficial effect may be related to the severity of liver disease, we conducted a sub-analysis on the basis of liver decompensation among these cirrhotic patients. The observed in-hospital mortality was virtually identical for both groups of patients with baseline compensated cirrhosis (93.2 % vs. 91.9 %, *p* = 0.830 by log-rank test; Fig. 2). However, the administration of prophylactic antibiotics showed significantly reduction of in-hospital mortality of patients with baseline decompensated cirrhosis compared to those without antibiotic prophylaxis (79.5 % vs. 65.6 %, *p* = 0.034 by log-rank test) after subgroup analysis (Fig. 3). The predictive risk factor associated with in-hospital death among decompensated cirrhotic patients was Rockall score (HR, 1.623; 95 % CI, 1.204–2.187; *p* = 0.001). Antibiotic prophylaxis has a protective role for in-hopital death in these patients (HR, 0.395; 95 % CI, 0.173–0.899; *p* = 0.027). For compensated cirrhotic patient, Rockall score (HR, 1.633; 95 % CI, 1.103–2.417; *p* = 0.014) and recurrent bleeding (HR, 3.684; 95 % CI, 1.040–13.05; *p* = 0.044) were the predictive factors associated with in-hospital death (Table 5).

**Table 5:** Univariate and multivariate analysis of potential risk factors for mortality with peptic ulcer bleeding after endoscopic treatment at different clinical stages of cirrhotic patients (subgroup analysis)

Variable	Compensated liver cirrhosis (*N* = 130)			Decompensated liver cirrhosis (*N* = 105)		
	Univariate analysis	Multivariate analysis		Univariate analysis	Multivariate analysis	
	*p*-value	Hazard ratio (95 % CI)	*p*-value	*p*-value	Hazard ratio (95 % CI)	*p*-value
Age	0.753			0.115		
Male gender	0.140			0.351		
Etiology of liver cirrhosis						
Alcoholic	0.136			0.639		
Viral hepatitis	0.281			0.422		
Cryptogenic	0.644			0.482		
Rockall score	0.014	1.633 (1.103–2.417)	0.014	0.001	1.623 (1.204–2.187)	0.001
MELD score	0.713			0.151		
Child-Pugh score	0.236			0.013	1.088 (0.855–1.384)	0.492
Other comorbidities						
Diabetes mellitus	0.394			0.477		
Hypertension	0.352			0.831		
CVD	0.729			0.195		
Stroke	0.168			0.450		
ESRD	0.986			0.128		
COPD	0.584			0.157		
Use of NSAID or aspirin	0.386			0.128		
Smoking	0.629			0.975		
Active alcoholism	0.533			0.177		
WBC (10^9^/l)	0.428			0.880		
Hb (g/dl)	0.921			0.463		
PLT (10^9^/l)	0.796			0.375		
PT (second)	0.898			0.597		
Creatinine (mg/dl)	0.766			0.869		
Albumin (g/l)	0.517			0.955		
Total bilirubin	0.450			0.167		
Blood units transfused	0.794			0.309		
Hypovolemic shock on admission	0.396			0.751		
High risk bleeding stigmata at ulcer base	0.593			0.676		
Time (h), bleeding to endoscopic treatment	0.734			0.085		
Combined treatment of endoscopic hemostasis	0.229			0.538		
Bacterial infection	0.327			0.224		
Recurrent bleeding	0.043	3.684 (1.040–13.05)	0.044	0.096		
Antibiotic prophylaxis	0.830			0.042	0.395 (0.173–0.899)	0.027

Abbreviations: *CI*, confidence interval; *COPD*, chronic obstructive pulmonary disease; *CVD*, Cardiovascular disease; *ESRD*, end-stage renal disease; *MELD*, Model for End-Stage Liver Disease; *NSAID*, nonsteroidal anti-inflammatory drug; *WBC*, white blood cells; *Hb*, hemoglobin; *PLT*, platelet count; *PT*, prothrombin time

## Discussion

Multiple factors contribute to the infection of cirrhotic patients with upper gastrointestinal bleeding. Cirrhotic patients usually have host defense defects against bacterial infection [16]. Furthermore, hypovolemia has been reported to increase intestinal bacterial translocation and depress reticuloendothelial system activity [17, 18]. In addition, patients with decompensated cirrhosis have more frequent episodes of infection than those with compensated liver disease [19]. Bacterial infection is associated with failure to control bleeding and patients with recurrent bleeding episodes [20]. An increase in portal pressure and changes in hemostasis induced by infection have been suggested as possible mechanisms [1, 21]. It has been suggested that intestinal bacterial translocation plays an important role in the pathogenesis of many infections. A meta-analysis of 12 trials comprising a total of 1241 patients with cirrhosis and gastrointestinal bleeding demonstrated that antibiotic prophylaxis significantly decreased the incidence of bacterial infections, re-bleeding, length of hospitalization, and mortality [22]. Currently, antibiotic prophylaxis is the standard care for cirrhotic patients with acute variceal bleeding but the prescription of antibiotic prophylaxis in cirrhotic patient with PUB has not been well documented. In order to address this particular issue in cirrhotic patients with PUB, we conducted this hospital-based cohort study for further clarification.

The results of the current study proved the benefit of antibiotic prophylaxis for bleeding peptic ulcers in cirrhotic patients with only 9 of the 88 patients (10.8 %) included in the antibiotic group developing bacterial infections during hospitalization. On the other hand, 37 of the 147 patients (25.2 %) in the control group suffered from a greater number of infectious events. In the multivariate analysis, higher Child-Pugh score, active alcoholism, and no antibiotic prophylaxis were significant predictors of infection development. Bacterial infections have been observed more frequently in Child-Pugh’s C patients than in those with Child-Pugh’s A or B [23] and active alcoholism can increase host susceptibility to bacterial infection, probably owing to the immunocompromised status following liver decompensation [24]. The bottom line is, the most common pathogens cultured in cirrhotic patients are gram-negative bacilli [15, 20]. The hypothesis behind this could be the onset of infection by intestinal bacterial translocation. In our study, gram-positive cocci accounted for 28.6 % of infections. This was probably because cirrhotic patients with upper gastrointestinal bleeding tended to receive multiple sessions of diagnostic and therapeutic invasive procedures, which in turn resulted in infections with gram-positive cocci [25, 26].

Overall, the ulcer rebleeding rate observed in the current study (22.3 %) which was higher than those reported in non-cirrhotic patients (3.2 %) [27] or in cirrhotic patients (7 %) [28]. The higher rebleeding events may be explained by the occurrence of a greater number of high risk bleeding stigmata identified in all of our patients with PUB (Forrest Ia, Ib: 51.9 % and Forrest IIa, IIb: 42.4 %) compared with those reported by the Marmo and Rudler groups [27, 28]. However, we observed that rebleeding and mortality do not seem to be associated with time to endoscopy. Although patients in the present study received endoscopy within 24 h, mortality remains high compared to patients without cirrhosis in the literature [29]. The predictors for rebleeding were: a higher Rockall score, more units of blood transfused, and no antibiotic prophylaxis. Again, current study proved the importance of administrating prophylactic antibiotics is crucial in preventing rebleeding in cirrhotic patients. Although presence of high risk bleeding stigmata is well-established risk factor for peptic ulcer bleeding, we didn’t find significantly correlation with rebleeding. Several factors might contribute to the discrepant results. First, it is possible that this retrospective analysis depended heavily on the completeness of the medical records and bias could exist. However, we reviewed endoscopic images or videos to determine the severity of the ulcer involved if uncertain chart description of ulcer morphology was encountered. Second, it was probably due to most ulcers belonged to high stigmata (Forrest Ia, Ib: 50.2 % and Forrest IIa, IIb: 38.7 %) in all of our enrolled patients. In fact, current guidelines do not recommend hemostatic therapy for patients with low-risk stigmata, such as those ulcers with a clean base (Forrest III) or ulcers with flat spots (Forrest IIc), and even recommend early discharge in selected patients with a low risk of rebleeding after endoscopic evaluation.

However, our study failed to show a significant difference in mortality or reduced length of hospital stay between patients who received prophylactic antibiotics and the control group. Probably, other factors such as requirement for mechanical ventilation, length of stay in intensive care unit, multiple organ dysfunction, and underlying comorbidities contributed to longer hospital stays and could be important determinants other than prophylactic antibiotic administration. Our study also clearly showed the increased mortality of patients with decompensated cirrhosis compared to those with compensated cirrhosis, with a quadrupling tripling of mortality risk (28.6 % vs. 7.7 %). Moreover, there was still an increased mortality of patients with decompensated cirrhosis compared with compensated cirrhosis even among patients with antibiotic prophylaxis (20.5 % vs. 6.8 %). Overall, the mortality rate in this study was 17 %. This was higher than those reported in previous studies of PUB [30]. This was not surprising because 44.7 % of our patients had decompensated cirrhosis. Patients with cirrhosis who develop PUB were at increased risk of mortality, and the risk appeared to increase further as patients progressed from compensated to decompensated cirrhosis. This could be due to the fact that patients with decompensated cirrhosis have an increased risk of developing sepsis, multiple organ failure, and death [31]. To our knowledge, our study was the first to compare the outcomes in cirrhotic patients with or without liver compensation who received antibiotic prophylaxis for PUB. Because no beneficial effect of antibiotic prophylaxis may be attributed to the severity of liver disease, we decided to perform a subgroup analysis on the basis of liver compensation by dividing our patients into 2 subgroups. Our results revealed an important message that intravenous ceftriaxone is only of benefit in reducing mortality in decompensated cirrhotic patients with peptic ulcer hemorrhage.

Mortality occurred in 13.6 % and 19 % of patients with or without antibiotic prophylaxis respectively. Only 20 % of the deaths were directly related to the bleeding episode, whilst the remaining deaths were associated with sepsis (*n* = 40, 25 %) and multi-organ failure (*n* = 22, 55 %). Despite substantial improvement in PUB management, the mortality in cirrhotic patients remains high, especially among patients with advanced liver cirrhosis [32, 33]. In a recently published study of 10,428 patients with non-variceal upper gastrointestinal bleeding in non-cirrhotic patients, death was associated with causes directly related to the bleeding episode in only 29 % of cases whilst in the remaining cases, co-morbidity played a fundamental role [34]. In other words, most PUB-linked deaths are not directly caused by the bleeding ulcer itself. This probably explains why routine antibiotic prophylaxis after gastrointestinal bleeding, even though it significantly reduces the incidence of bacterial infections, does not significantly affect the mortality rate. As most mortality occurs as a result of multi-organ failure, this suggests that improved treatments and supportive care should be provided to prevent sepsis complications and key organ failure. On the other hand, three patients died from uncertain causes soon after endoscopy and they were excluded from study. The causes of mortality could not be determined whether they were procedure-related (suffocation, aspiration pneumonia, bowel trauma, incomplete endoscopic hemostasis, as extra), bleeding-related or other causes such as acute myocardial infarction, brain stroke, perforated peptic ulcer, as extra. Therefore, we excluded patients who die within 24 h due to unable to analyses the cause of mortality.

The Rockall score was found to be an independent predictor of in-hospital mortality [11]. It makes séance as it includes both clinical and endoscopic criteria to predict the risk of rebleeding and death. The bottom line is, we used the MELD score, the Child-Pugh classification, and the Rockall’s risk scoring system to assess our patients but mortality was surprisingly not related to the severity of liver dysfunction as expressed by either the MELD score or the Child-Pugh classification. Only Rockall scores were associated with rebleeding and death. The effectiveness of the Rockall score as a predictor of in-hospital death in cirrhotic patients with PUB proved to be similar to that reported in non-cirrhotic patients [35].

A major strength of this study is that it was a well-defined consecutive registered study cohort treated by a standard endoscopic intervention. Patients who received antibiotic prophylaxis were defined as antibiotic group and those who not received were used as historical control. To provide the highest possible level of uniformity and to minimize differences in the entry, only patients who received their initial and subsequent treatment in our unit were studied. The robustness of this study is enhanced by the restriction of subjects to patients without an apparent source of infection at first bleeding episode, strict exclusion criteria and the complete follow-up of the cohort. The use of rebleeding, infection and death as the main outcomes provided consistent and objective endpoints in the study. Unlike other studies, which have included patients at differing disease stages without separate analyses, our study did evaluate the impact of liver function on the efficacy of antibiotics.

Current study encountered some limitations. First, the sample size was small and a single center cohort study. Certain selection biases could exist and must be cautious in extrapolating the results. Multicenter data with a larger sample size is mandatory. Second, the authors also served as data abstractors and could have been biased because they were not blinded which was not possible in this retrospective cohort study. Therefore, exposure bias might be considered a limitation of this study. Third, survivor bias may have occurred in which surviving patients who probably had more opportunities to receive intensive care, such as ventilator support, hemodialysis, repeated endoscopic hemostasis and more blood product transfusions.

Those patients who belonged to control group were enrolled between January 1, 2008, and December 31, 2009. The antibiotic group has been routine practice to prescribe prophylactic antibiotics to cirrhotic patients with gastrointestinal bleeding in our hospital since 2010. This minimized bias in our results, because both cohorts were enrolled according to identical treatment protocol adhering to our hospital policy in our daily practices for PUB population even though during the different period.

## Conclusions

This study confirmed the beneficial effects of antibiotic prophylaxis in patients with cirrhosis following APU with the reduced infections and rebleeding events. The study also suggests that antibiotic prophylaxis could improve survival in decompensated cirrhotic patients following acute PUB. The Rockall score was the strongest predictive factor of in-hospital mortality. Further studies directed to explore ways to improve the overall outcome of cirrhotic patients with PUB are mandatory.

## Abbreviations

PUB: Peptic ulcer bleeding
NSAID: Non-steroid anti-inflammatory drugs
HBsAg: Hepatitis B antigen
Anti-HCV: Hepatitis C antibody
HR: Hazard ratio
EV: Esophageal varices
GV: Gastric varices
MELD: Model for end-stage liver disease
ESRD: End stage renal disease
COPD: Chronic obstructive pulmonary disease
